# The pattern of comorbidities in cancer patients in Lagos, South-Western Nigeria

**DOI:** 10.3332/ecancer.2018.843

**Published:** 2018-06-13

**Authors:** Omolola Salako, Paul T Okediji, Muhammad Y Habeebu, Omolara A Fatiregun, Opeyemi M Awofeso, Kehinde S Okunade, Ifedayo A Odeniyi, Kahmil O Salawu, Evaristus O Oboh

**Affiliations:** 1 Department of Radiotherapy, Lagos University Teaching Hospital, Idi-Araba, Lagos 100254, Nigeria; 2 Research and Development, Sebeccly Cancer Care, Yaba, Lagos 101212, Nigeria; 3 Department of Radiotherapy, Lagos State University Teaching Hospital, Ikeja, Lagos 100254, Nigeria; 4 College of Medicine, University of Lagos, Akoka, Lagos 100254, Nigeria; 5 Department of Obstetrics and Gynaecology, College of Medicine, University of Lagos, Lagos 100254, Nigeria; 6 Department of Medicine, Faculty of Clinical Sciences, College of Medicine, University of Lagos, Lagos 100254, Nigeria; 7 Department of Radiotherapy, University of Benin Teaching Hospital, Benin 300283, Nigeria

**Keywords:** cancer, comorbidities, Charlson comorbidity index, Lagos, Nigeria

## Abstract

**Purpose:**

Comorbidities have been indicated to influence cancer care and outcome, with strong associations between the presence of comorbidities and patient survival. The objective of this study is to determine the magnitude and pattern of comorbidities in Nigerian cancer populations, and demonstrate the use of comorbidity indices in predicting mortality/survival rates of cancer patients.

**Methods:**

Using a retrospective study design, data were extracted from hospital reports of patients presenting for oncology care between January 2015 and December 2016 at two tertiary health facilities in Lagos, Nigeria. Patient comorbidities were ranked and weighted using the Charlson comorbidity index (CCI).

**Results:**

The mean age for the 848 cancer patients identified was 53.9 ± 13.6 years, with 657 (77.5%) females and 191 (22.5%) males. Breast (50.1%), cervical (11.1%) and colorectal (6.3%) cancers occurred most frequently. Comorbidities were present in 228 (26.9%) patients, with the most common being hypertension (20.4%), diabetes (6.7%) and peptic ulcer disease (2.1%). Hypertension-augmented CCI scores were 0 (15.6%), 1–3 (62.1%), 4–6 (21.7%) and ≥7 (0.6%). The mean CCI scores of patients ≤50 years (0.8 ± 0.9) and ≥51 years (3.3 ± 1.2) were significantly different (*p* < 0.05). Patients with lower mean CCI scores were more likely to receive chemotherapy (2.2 ± 1.6 versus 2.5 ± 1.9; *p* < 0.05) and/or surgery (2.1 ± 1.5 versus 2.4 ± 1.7; *p* < 0.05).

**Conclusion:**

Comorbidities occur significantly in Nigerian cancer patients and influence the prognosis, treatment outcome and survival rates of these patients. There is a need to routinely evaluate cancer patients for comorbidities with the aim of instituting appropriate multidisciplinary management measures where necessary.

## Introduction

Cancer is one of the most commonly diagnosed conditions globally, and is also a leading cause of death worldwide. The increasing cancer burden in developing countries such as Nigeria is a significant public health problem that governments are grappling with. The GLOBOCAN 2012 report showed that there were 14.1 million new cases of cancer, 8.2 million mortalities from cancer and 32.6 million people living with cancer worldwide in 2012 [1]. Of these huge figures, the major morbidity and mortality burden are borne by developing countries as 70% of deaths from cancer occur in middle- and low-income nations [2]. With more Nigerian patients presenting to cancer clinics and the increasing availability of cancer care, oncologists are managing more patients with cancer and comorbidities. Bellizzi and Rowland [3] reported that 69–88% of cancer patients have at least one comorbidity. As a result of this, the treatment of such patients becomes convoluted and its impact on survival and quality of life becomes significant.

Comorbidities are medical conditions that coexist with the disease of interest, but are not related in causality or aetiology to the primary diagnosis [4]. They may occur prior or at the same time as the primary disease. For several years, the influence of these coexisting medical conditions on the outcome of care for the cancer patient has often been ignored. According to Piccirillo and Feinstein [5], a large number of the most frequently used cancer classification systems in clinical practice do not consider critical patient-based prognostic factors such as the general health of the cancer patient—which is dependent on the presence, number and pathophysiological severity of any coexisting illnesses or conditions. These illnesses or conditions that are not as a result of the adverse effect of therapy for cancer, which exist before the cancer diagnosis was made, are referred to as comorbidities [6].

The importance of comorbidities in cancer patients draws from an increasing awareness of their impacts on cancer care and outcome. Recent research by Lund *et al* [7], Patniak *et al* [8] and many other authors show that a high level of comorbidity correlates with reduced survival indices of patients with cancer. This can be explained by the increase in mortality caused by the presence of the comorbid conditions. Moreover, the increase in cancer-specific mortality as a result of these comorbid conditions have also been attributed to the use of suboptimal antineoplastic treatment regimens [9, 10], and/or an increase in treatment toxicity leading to reduced treatment compliance [11]. In addition, toxicity arising from cancer therapy can also influence the outcome of care. The presence of at least one comorbid condition has been associated with diagnostic dilemmas and delays in making cancer diagnosis, leading to more advanced disease at the time of final diagnosis [12]. Other effects of multiple comorbidities on cancer patients include an increase in the risk of complications from surgery [13], higher rates of postoperative mortality [14–16], and a greater consumption of medical resources [17].

One of the factors associated with an increasing incidence of cancer worldwide is the increase in life expectancies [18]. Considering that the prevalence of most noncommunicable diseases rises with age, it is easy to understand the increasing prevalence of coexisting medical disorders in cancer patients over their lifespan [19]. Also, advancement in medical therapies has also contributed to the doubling of the prevalence of chronic diseases between 1985 and 2005, and a threefold increase in the proportion of patients with greater than four coexisting medical conditions [20]. In addition, comorbidity has been associated with individuals who have low social support, mental health disorders and high levels of socioeconomic deprivation [21]. This explains in part why patients in developing countries with higher levels of socio-economic deprivation are at greater risk of multiple comorbidities. Furthermore, since comorbidity has been associated with a significant drop in functional reserves and an increased physical frailty, it suggests the reasons why cancer patients in developing countries like Nigeria experience higher levels of treatment-related toxicities and also have suboptimal outcomes [22].

Nigeria has a huge burden of cancer patients, as estimates show that over 102,000 Nigerians are diagnosed with cancer annually, and 5-year prevalence rates of breast, cervical, prostate and colorectal cancer have been put at 37.7%, 15.4%, 13.4% and 3.7%, respectively [1, 23]. In addition to this high cancer prevalence, chronic nonmalignant illnesses are also very common as studies have identified significant prevalence rates of illnesses such as diabetes (4.0%), cardiovascular disease (12%), bronchial asthma (14–18%) and others amongst the Nigerian population [24–28]. The World Health Organization estimates the mortality from noncommunicable chronic illnesses to be 818/100,000 of males and 806/100,000 of females in 2012 [29]. There is presently a dearth of information about the prevalence and effects of comorbidities on cancer patients in Nigeria. Considering the established effects of comorbidities on cancer care and outcome, it becomes necessary to understand with greater accuracy the magnitude and pattern of comorbidities in Nigerian cancer patients.

Therefore, the general focus of this research is to determine the magnitude and pattern of comorbidities in the Nigerian cancer population, and to elucidate the demographic and social factors that are most responsible for the occurrence of these comorbidities in cancer patients.

## Methodology

### Patient selection

This retrospective study focused on all patients with various types of cancers who presented at the Departments of Oncology and Radiotherapy of the Lagos University Teaching Hospital (LUTH), Idi-Araba, and the Lagos State University Teaching Hospital, Ikeja, for treatment between January 2015 and December 2016. These two public tertiary centres receive the bulk of patients with cancers in Lagos State, surrounding states and the West-African sub-region. Eligible patients were selected on the basis of these criteria: (a) clinical and/or histological diagnosis of a malignancy, (b) ages older than 18 years and (c) diagnosed or treated for a malignancy within the study time frame. Patients with skin cancers and clinical records with missing or unknown data were excluded.

### Data collection

Data were collected from the hospital records of patients who fit into the eligibility criteria. Emphasis was on patient demographics (such as age, hospital number, gender, occupation, religion, marital status and contact information), clinical characteristics (such as type of cancer, histology, grade, stage and presence of comorbidities) and social history. Information about comorbidities was recorded using the Charlson comorbidities index (CCI), which has been validated and tested with huge success [30, 31]. The index was based on weighted measures of 18 different comorbidities (excluding solid tumours) and calculated by adding one point for each decade of life after 40 years—one point for those between 41 and 50 years, two points for 51–60 years, three points for 61–70 years and four points for those ≥71 years. A score of one point for hypertension was added to the index comorbidities, particularly because of the presumed high prevalence of the disease in many cancer patients, and the recent modifications of the CCI to include hypertension (hypertension-augmented CCI) [32]. Presence of comorbidities was determined by past medical history or present history of any of the index illnesses. Data quality was ensured for accuracy and completeness by the use of well-trained medical personnel and review of collected data by a different independent review team. Histological diagnosis was based on reports written by verified and certified histopathologist from within and outside the study centres.

### Data analysis

All statistical analyses were conducted within the software platform of the IBM Statistical Package for the Social Sciences (SPSS) for Windows, version 20.0. Basic demographic, clinical and social data were presented and analysed using descriptive statistics in the form of frequency tables. Continuous data including CCI scores were presented in the form of means ± standard deviation. Associations between mean CCI and treatment options were determined using independent t-tests. Statistical significance (*p*-values) set at ≤0.05.

### Ethical considerations

Ethical approval for this research was obtained from the Health Research and Ethics Committee of the LUTH, Idi-Araba, and carried out in line with the principles of the Helsinki Declaration. Consideration was made for the confidentiality of data and protection of all data obtained during the course of this research.

## Results

The analysis included 848 patients who fulfilled the eligibility criteria with various types of cancers seen between January 2015 and December 2016. The mean age was 53.9 ± 13.6 years with 191 (22.5%) males and 657 (77.5%) females (Table 1). The majority of the patients were managed for breast cancer (425, 50.1%).

Breast, cervical, colorectal, prostate and ovarian cancers occurred more often in descending order of frequency, and all together, these five cancers constituted about three-quarters of the patient population (Table 1). Comorbidities were present in 228 (26.9%) patients and the most commonly used treatment option was chemotherapy (687, 81.0%). Surgical treatments and radiotherapy were administered to 380 (44.8%) and 332 (39.1%), respectively.

The calculation of the total CCI is presented in Table 2. Each decade after 40 years of age was scored one incremental point till ≥71 years. Hypertension was the most prevalent comorbidity occurring in 173 (20.4%) of patients. This is followed by diabetes mellitus and peptic ulcer disease.

On the basis of CCI scores, Table 3 shows the proportions of the various scores ranging between 0 and 9. The bulk of the patients lie between scores 0 and 4. Patients with CCI score of 1 have 1.45 times the probability of dying within 1 year, which increases to 4.4 times and above for patients with CCI scores of 4 and above.

Patients with prostate cancer had the highest mean CCI scores (3.7 ± 1.2), followed by lung cancer (3.4 ± 1.2) and pancreatic cancer (3.0 ± 1.2). These same set of patients had the highest mean ages in decreasing order—66.5, 66.2 and 61.4 years for prostate, lung and pancreatic cancer, respectively (Table 4). Breast cancer patients had some of the lowest CCI scores with a mean of 2.0 ± 1.5 and a mean age of 52.4 ± 12.7 years.

**Figure 1.**
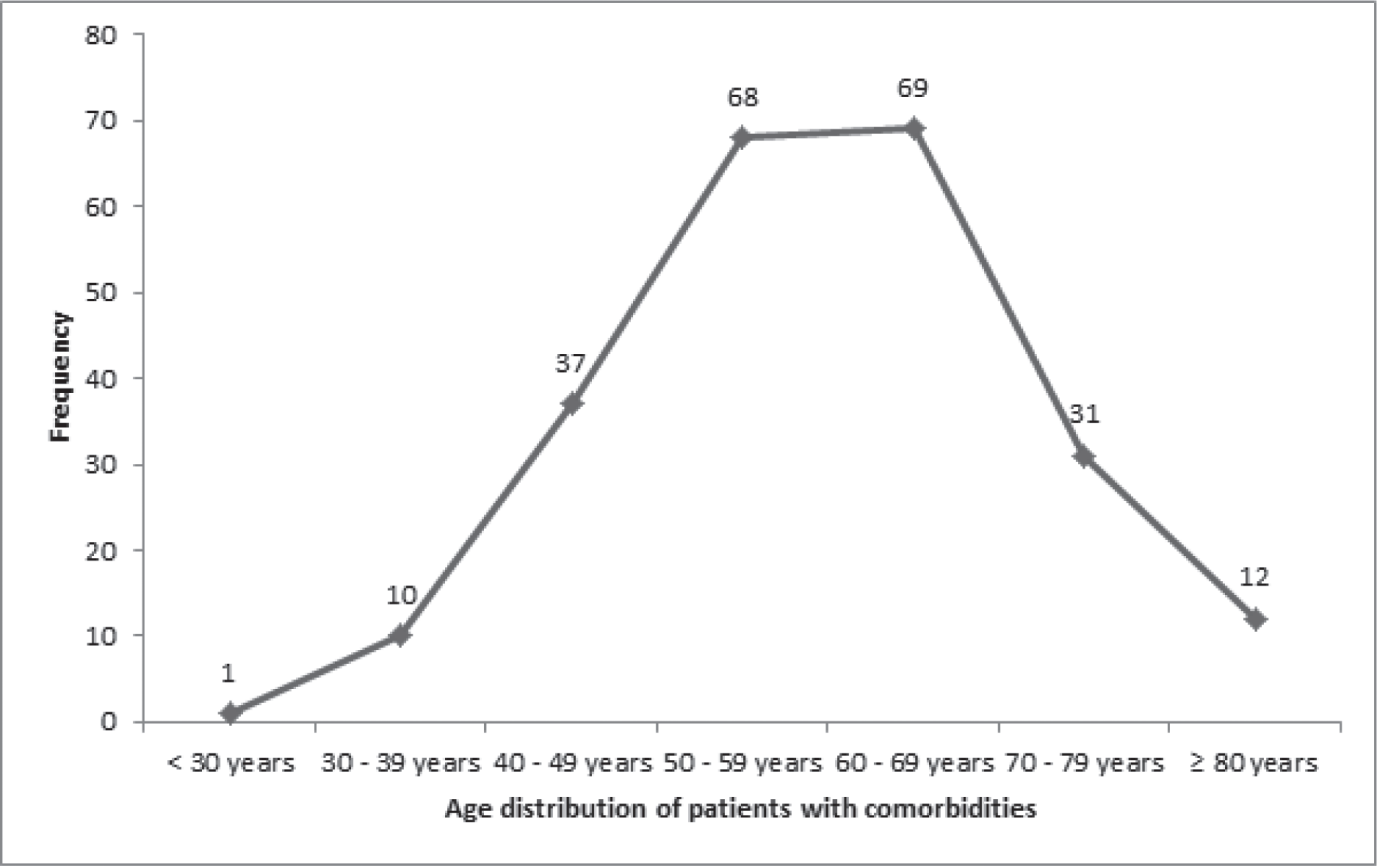
Age distribution of comorbidities in the study population.

Patients with ages 50 years or less had a significantly lower mean CCI (0.8 ± 0.9) than those who are at least 51 years or more (*p* < 0.05; Table 5). As the ages increased, the chances of developing a comorbid illness increased, with a peak between the ages of 60 and 69 years (Figure 1). There were also significant differences in the mean CCIs of patients who either received chemotherapy compared to those that did not (*p* = 0.05), and between those who received surgical treatments and those who did not (*p* = 0.01). However, there was no significant difference in the mean CCIs of patients who did or did not receive radiotherapy for their primary disease (*p* = 0.52).

## Discussion

The CCI has been used extensively to assess comorbidity burden across populations [33], assess risk of mortality in groups of patients [10], predict costs of treating chronic illnesses [17, 34], and predict a variety of malignancies and other clinical outcomes. From the results obtained, about a quarter of the patients (26.9%) had at least a comorbid illness. Studies have pointed out a wide range of comorbidities in cancer patients, from as low as 0.4% to as high as 90% [35, 36]. This huge variability in the prevalence of comorbidities suggests that the prevalence of comorbidity may be influenced by specific factors such as age, geographical location, race/ethnicity and socioeconomic status.

The influence of age on the prevalence of comorbidity is demonstrated by a Nigerian study conducted on 249 elderly patients in Zaria which showed that 38.2% of the patients had comorbid conditions [37]. This is much higher than the 26.9% obtained in this study, and this may be due to the fact that just 58.0% of the patients in this study were older than 50 years, indicating a significant population of younger patients in this study as compared with those in the Zaria study in which over 90% were older than 50 years. Studies focused on older patients tend to report a higher level of comorbidity while those based on administrative data or medical notes in which a wider range of ages are found tend to present a lower prevalence of comorbidity [36]. Unsurprisingly, a significant difference in the mean CCI scores between patients ≤50 years and those >50 years (*p* < 0.05) was found in this study. This implies that patients who are at least 50 years must be evaluated for the presence of comorbidities at presentation for a malignancy.

In close relation to the effect of age on the prevalence of comorbidity is the distribution of comorbidities as the most common comorbidity experienced by these patients was hypertension (173, 20.4%). This was followed by diabetes mellitus and peptic ulcer disease in 53 (6.2%) and 18 (2.1%) patients, respectively. In the study by Adewuyi *et al* [37], hypertension was the most common comorbidity found amongst elderly cancer patients, and followed by diabetes mellitus. This was not surprising as both hypertension and diabetes are chronic conditions that are commonly found among elderly individuals [38]. While both conditions are not usually life threatening in the short term, their co-occurrence with a malignancy alters the prognosis and indicates the need for more specialised care, and especially, a multidisciplinary approach to care [31, 36, 37].

Also, the type of cancer has been found to influence both the presence and magnitude of comorbidity. In this study, prostate cancer had the highest mean CCI with 3.7 ± 1.2. Breast cancer, which was the most prevalent type of cancer, occurring in over half of the patients, had a mean CCI of 2.0 ± 1.5. In between were lung (3.4 ± 1.2), pancreas (3.0 ± 1.2) and endometrial (2.7 ± 1.3) cancers. Edwards *et al* [39] had previously reported that patients with prostate and breast cancer tend to have similar levels of comorbidity while lung cancer patients had a much greater level of comorbidity. Using risk factors, they had concluded that cancers strongly associated with chronic risk factors such as smoking were at one end of the comorbidities spectrum while cancers less (or inversely) associated with risk factors (e.g. prostate and breast cancers) were at the other end of the spectrum. This does not appear to be so from the results of this study, in which prostate and breast cancers were on different ends of the comorbidities spectrum. This is likely due to the influence of age, as the mean age for patients with prostate cancer was 66.5 ± 8.7 years as against 52.4 ± 12.7 years for those with breast cancer.

In the past, studies have suggested that the presence of comorbidities determine treatment choices. According to Sarfati *et al* [36], patients who have significant comorbidities are generally less likely to be offered curative treatment for their primary malignant conditions than those without. For instance, it has been shown that the offer and uptake of chemotherapy in patients with colorectal cancer is relatively lower in those who have comorbidities regardless of age [40–42]. This was found to be true in this study as mean CCIs of those who did not receive chemotherapy was significantly higher than those who were given chemotherapy. This implies that patients with less number of comorbidities were more likely to be given chemotherapy. The same significant difference was obtained for surgical treatment, although the relationship between comorbidities and surgical treatment is less clear as some studies have reported no association while others suggest an inverse relationship between the presence or level of comorbidity and surgery [36, 43, 44]. For radiotherapy, there was no statistically significant difference in the mean CCI scores of those who received radiotherapy and those who did not.

A number of reasons have been proposed to explain the influence of comorbidity on treatment choices, offering and uptake. It has been shown that the toxicity and side effects of chemotherapy and radiotherapy may be increased with the presence of co-existing illnesses [36]. Other clinicians have suggested that the life expectancy of cancer patients worsened by comorbidities is not sufficient to justify the use of curative but potentially toxic treatment options [40, 45–47]. Even though there have been clear associations between the presence of comorbidity and the choice of chemotherapeutic/surgical treatments in this study, this is not as clear cut in oncology clinics as significant inconsistencies in the determination of treatment options for cancer patients might have contributed to the differences rather than a simple consideration of the extent or presence of a comorbidity. Nonetheless, not all patients with significant comorbidities or advanced age should be excluded from more aggressive cancer treatment options they desperately need.

Many studies have related CCI scores with the risk of mortality in cancer patients. Comorbidities worsen the quality of life of patients with cancer, and also increase their chances of dying earlier [48–50]. The CCI has been shown to be a very strong and accurate predictor of mortality within 1, 5 and 10 years. On the basis of this, it is clear that the majority of these patients have a significant relative risk of death, ranging from 1.45 to 13.37. Since the bulk of the patients have CCI scores between 1 and 3, their 10-year survival rates would range from as low as 45% to as high as 73%. Also, the association between CCI scores and mortality has been linked to associations with quality of life and performance status (PS) [48–52]. Mayr *et al* [52] found that the CCI and Eastern Cooperative Oncology Group (ECOG) were strong predictors for postoperative mortality in bladder cancer. In future studies, it will be interesting to see a direct correlation between the CCI and PS indices such as the ECOG or the Karnofsky scale considering that they are standard indices in clinical practice and research for quantifying the aggregate impact of comorbidities on the quality of life of cancer patients.

While considering the significant frequency of comorbidities noted in the population studied, and the strength of association between comorbidities and treatment options, it is important to note some of the limitations of this study. This study utilised a retrospective design which makes it difficult to estimate treatment outcomes and overall survival like it has been done in some other studies. This also made it difficult to evaluate the relationships between common comorbidities such as hypertension or diabetes and survival. It is also understood that the severity of each comorbid condition determines how it affects the cancer patient, more so, that the effect of a comorbid condition varies across cancer types and treatments.

## Conclusion

A significant proportion of cancer patients in Lagos suffer from one or more comorbidities which influences their treatment and overall clinical outcomes. Hence, there is a need to evaluate cancer patients for comorbidities with the aim of instituting appropriate multidisciplinary management measures where necessary.

## Disclaimer

The views expressed in the submitted article are solely our own and not an official position of any institution or funder.

## Figures and Tables

**Table 1.** Characteristics of the study population.

	Frequency (*n* = 848)	Percentage of total population (%)
**Age**	Mean: 53.9 ± 13.6 years	
**Gender**		
**Male**	191	22.5
**Female**	657	77.5
**Type of cancer**		
**Breast**	425	50.1
**Cervical**	94	11.1
**Colorectal**	53	6.3
**Prostate**	41	4.8
**Ovarian**	22	2.6
**Others**	213	25.1
**Comorbidities**		
**Present**	228	26.9
**Absent**	620	73.1
**Treatment characteristics**		
**Chemotherapy**		
**Yes**	687	81.0
**No**	161	19.0
**Radiotherapy**		
**Yes**	332	39.1
**No**	516	60.9
**Surgery**		
**Yes**	380	44.8
**No**	468	55.2

**Table 2.** Distribution of comorbidities based on the age-adjusted hypertension-augmented CCI, *n* = 848.

Attribute	Frequency	Percentage
**Myocardial infarction**	5	0.6
**Congestive heart failure**	9	1.1
**Peripheral vascular disease**	2	0.2
**Cerebrovascular disease**	2	0.2
**Dementia**	2	0.2
**Chronic pulmonary disease**	4	0.5
**Rheumatic disease**	0	0.0
**Peptic ulcer disease**	18	2.1
**Mild liver disease**	2	0.2
**Diabetes mellitus without end-organ damage**	53	6.2
**Diabetes mellitus with end-organ damage**	4	0.5
**Hemiplegia**	4	0.5
**Renal disease**	2	0.2
**Lymphoma**	7	0.8
**Leukaemia**	2	0.2
**Moderate liver disease**	2	0.2
**Acquired immunodeficiency syndrome**	5	0.6
**Hypertension**	173	20.4

**Table 3.** Distribution of CCI scores among cancer patients with their relative risks of death within 1 year (relative risk figures from *Charlson et al* [30]).

CCI score	*n* = 848 (%)	Estimated relative risk of death within 1 year	95% confidence interval
**0**	132 (15.6)	1.00	–
**1**	183 (21.6)	1.45	1.25–1.60
**2**	175 (20.6)	2.10	1.57–2.81
**3**	169 (19.9)	3.04	1.96–4.71
**4**	114 (13.4)	4.40	2.45–7.90
**5**	56 (6.6)	6.38	3.07–13.2
**6**	14 (1.7)	9.23	3.84–22.20
**7**	3 (0.4)	13.37	4.81–37.22
**9**	2 (0.2)	–	–

**Table 4.** Distribution of cancer types by mean CCI scores and mean ages.

Type of cancer	Mean CCI score	Age
**Prostate**	3.7 ± 1.2	66.5 ± 8.7
**Lung**	3.4 ± 1.2	66.2 ± 10.8
**Pancreas**	3.0 ± 1.2	61.4 ± 12.0
**Endometrial**	2.7 ± 1.3	58.5 ± 9.9
**Cervical**	2.4 ± 1.4	56.0 ± 12.6
**Liver**	2.2 ± 1.7	53.9 ± 13.8
**Colorectal**	2.2 ± 1.6	52.8 ± 13.9
**Breast**	2.0 ± 1.5	52.4 ± 12.7
**Nasopharyngeal**	1.8 ± 1.4	48.7 ± 14.8
**Ovarian**	1.5 ± 1.4	51.2 ± 16.5

**Table 5.** Relationships between mean CCI scores and age or treatment options.

Variable	*n*	Mean CCI	*p*-value
**Age**			
**≤50 years**	356	0.8 ± 0.9	0.00001*
**≥51 years**	492	3.3 ± 1.2	
**Chemotherapy**			
**No**	161	2.5 ± 1.9	0.05*
**Yes**	687	2.2 ± 1.6	
**Surgery**			
**No**	468	2.4 ± 1.7	0.01*
**Yes**	380	2.1 ± 1.5	
**Radiotherapy**			
**No**	516	2.2 ± 1.6	0.52
**Yes**	332	2.3 ± 1.6	

* Significant *p*-values
